# Implementing pragmatic case finding to address alcohol use in general practice: a mixed methods feasibility study

**DOI:** 10.1080/02813432.2025.2598835

**Published:** 2025-12-19

**Authors:** Sebastian Potthoff, Håvar Brendryen, Haris Bosnic, Rashmi Bhardwaj-Gosling, Kristina Riis Iden, Anne Lill Mjølhus Njå, Amy O’Donnell, Torgeir Gilje Lid

**Affiliations:** ^a^ Centre for Alcohol and Drug Research, Stavanger University Hospital, Stavanger, Norway; ^b^ School of Communities and Education, Faculty of Health and Wellbeing, Northumbria University, Newcastle Upon Tyne, United Kingdom; ^c^ Department of Psychology, Faculty of Social Sciences, University of Oslo, Oslo, Norway; ^d^ Norwegian Reading Centre, Faculty of Arts and Education, University of Stavanger, Stavanger, Norway; ^e^ Faculty of Health Sciences and Wellbeing, University of Sunderland, Sunderland, United Kingdom; ^f^ Population Health Sciences Institute, Faculty of Medical Sciences, Newcastle University, Newcastle, United Kingdom; ^g^ Faculty of Health Sciences, University of Stavanger, Stavanger, Norway; ^h^ Research Unit for General Practice, NORCE Norwegian Research Centre, Bergen, Norway

**Keywords:** Pragmatic case finding, alcohol interventions, general practice, primary care, tailored implementation

## Abstract

**Background:**

Screening and brief interventions (SBIs) for alcohol use are effective but challenging to implement in primary care settings. Universal screening is resource-intensive and may not align with general practitioners’ (GPs) perceived professional role. Pragmatic case finding (PCF), which integrates alcohol discussions into clinically relevant contexts, may provide a feasible alternative to traditional SBI.

**Aim:**

This study aimed to assess the feasibility and acceptability of tailored, theory-based educational outreach visits (EOVs) to embed PCF in primary care, explore its influence on professional practice in addressing alcohol, and examine changes in determinants of GP behaviour pre- and post-implementation.

**Design and setting:**

Four EOVs were delivered in GP clinics in Stavanger and Oslo, Norway, involving 37 GPs and 22 support staff, to enhance GPs’ ability to manage alcohol-related health problems.

**Method:**

A mixed-methods feasibility study comprising semi-structured group interviews and quantitative surveys. Group interviews explored GPs’ experiences, while the Determinants of Implementation Behaviour Questionnaire (DIBQ) assessed changes in knowledge, skills and intentions. Qualitative data were thematically analysed. Quantitative data were analysed using descriptive statistics.

**Results:**

GPs (*n* = 10) perceived the EOVs as feasible and acceptable, preferring in-person over remote delivery. Key themes included greater awareness of alcohol’s health impacts, sustaining awareness of hidden cases, reducing stigma through normalised discussions, and balancing motivation with the challenge of changing entrenched habits. Survey findings (*n* = 19) showed a gradual, positive shift in GPs’ knowledge, skills, and goals to discuss alcohol.

**Conclusion:**

The EOVs were feasible and acceptable for embedding PCF in primary care. They may strengthen GPs’ capacity to address alcohol in routine consultations, but further research is needed to assess fidelity, sustainability, and patient-level outcomes.

**Trial registration number:**

ClinicalTrials.gov ID: NCT04725552.

## Introduction

Alcohol screening and brief intervention (SBI) is proven to be effective at reducing alcohol consumption amongst at-risk drinkers and has the potential to reach a large proportion of the population at relatively low cost [1,2]. However, there are many barriers to widespread implementation of SBI in primary health care, including at an individual level (practitioner or patient), organisational level (practice) and societal level (regulations, cultural factors) [3–9]. General practitioners (GPs) may fear alienating their patients by raising a potentially sensitive topic in routine consultations, even though they see addressing alcohol-related health problems as their responsibility [10,11]. So far, large-scale implementation studies on SBI have not been successful, and while brief alcohol interventions (BAIs) are effective, challenges remain regarding identifying which patients should receive these interventions [7,8,12–14].

The suboptimal implementation of potentially effective BAIs in clinical practice is an increasing concern, and strategies to reduce this research–practice gap, such as research translation centres, have recently been launched in England and Australia [15]. While this gap may be perceived as primarily a lack of translation from research to practice, there is an equally important need for translation from practice to research. As four decades of research on SBI has documented, the task of screening and offering BAIs to those with hazardous or harmful alcohol consumption may often be at odds with both the patient’s agenda and the GP’s understanding of their responsibility [14,16–18].

To date, most studies on SBI have focused on universal, widespread screening, in part due to evidence that clinical judgment alone may not be sufficient to identify patients in need of alcohol advice [19]. Relatively few studies have explored pragmatic (targeted or relevance-based) strategies to identify patients that might benefit from BAIs [20,21], despite research to suggest they may be more acceptable to GPs [19]. Moreover, such approaches may be more clinically useful as even though universal screening identifies more risk drinkers, targeted approaches yield a higher prevalence of at-risk alcohol consumers [21]. Several strategies based on clinical relevance have been reported, e.g. *semi-systematic method, pragmatic case finding,* and *relevance criteria* [10,20,22]. Pragmatic case finding (PCF) is a strategy based on clinical relevance, where the practitioner addresses alcohol when it might be relevant to the condition that the patient is presenting with, either as cause, complicating factor or due to increased vulnerability [10]. PCF is more compatible with GPs’ skills and clinical reasoning and may potentially improve clinical practice [23].

PCF relies on GPs’ clinical judgement, and its acceptability and uptake are likely to vary with practitioners’ skills, experience, and confidence. A recent systematic review using the Theoretical Domains Framework (TDF) and Behaviour Change Wheel (BCW) identified common barriers to implementing SBI in primary care, including limited knowledge and skills, time constraints, uncertainty about professional role, and perceived patient discomfort in discussing alcohol [24]. The Capability-Opportunity-Motivation-Behaviour (COM-B) model, which is at the core of the BCW, provides a structure for understanding these determinants, while the TDF offers more detailed mapping to COM-B domains [25,26]. The BCW and associated Behaviour Change Techniques Taxonomy provide practical guidance for selecting intervention components—such as educational outreach visits (EOVs) to enhance capability, peer discussions to strengthen motivation, and clinical tools to increase opportunity [27]. Together, these perspectives highlight implementation challenges for SBI and informed the design of our strategy to embed PCF in practice.

Alongside the value of theory-based approaches, both implementation content and implementation strategies should be tailored to the relevant context, which requires a continuous engagement of stakeholders in planning, execution and evaluation of the implementation efforts [28]. For example, GPs in Norway work primarily in self-owned group practices, where every citizen is listed with a specific GP. Public trust in this system has been very high, with 99.8% of the population listed with a GP [29]. Since 2017, there has been an ongoing crisis in primary care due to increased workload and low recruitment. By the end of 2021, one quarter of the lists were staffed with locums, and 138.453 inhabitants had no access to a GP [30]. This situation complicates both implementation of new practices and the possibility of doing research in GP clinics.

This feasibility study had three aims: (1) to assess the feasibility and acceptability of tailored, theory-based EOVs designed to embed PCF in primary care; (2) to explore how participation in the EOVs influenced GPs’ professional practice in addressing alcohol; and (3) to examine changes in GP-reported determinants of behaviour (COM-B/TDF domains) before and after the EOVs.

## Methods

### Design and setting

**Figure 1.**
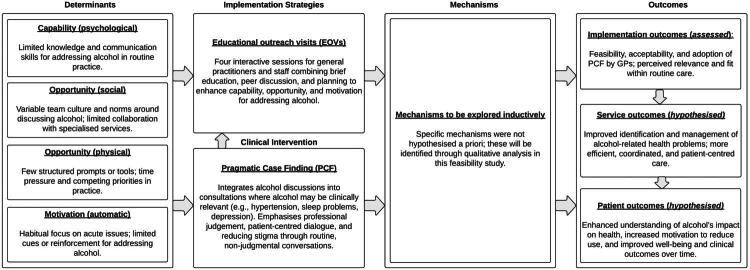
Initial Implementation Research Logic Model [32] showing hypothesised relationships between determinants, implementation strategy (Educational outreach visits; EOVs), the clinical intervention (Pragmatic Case Finding; PCF), mechanisms of impact, and outcomes. Determinants and strategies were adapted from Potthoff et al. [33], PCF from Lid et al. [10,34]. Mechanisms of impact were explored inductively in this study. Service- and patient-level outcomes are hypothesised for future evaluation.

**Figure 2.**
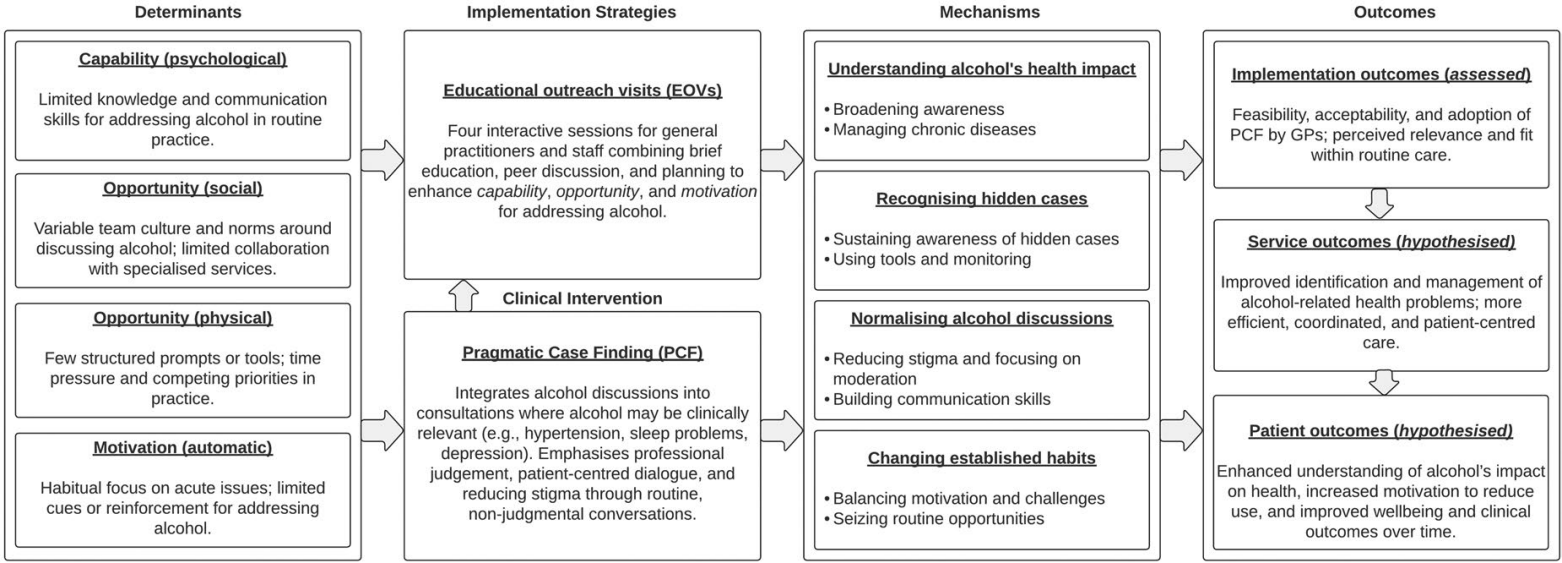
Empirically identified mechanisms of impact from the qualitative analysis were integrated into the Implementation Research Logic Model (IRLM) introduced in Figure 1. The model illustrates how EOVs may have supported implementation of PCF in general practice.

This feasibility study used a mixed-methods approach including semi-structured interviews and a theory-based survey. We developed an initial logic model (Figure 1) using the Implementation Research Logic Model (IRLM) framework [31] to illustrate how EOVs were expected to support implementation of PCF in general practice. The model specifies the planned relationships between determinants, implementation strategy (EOVs), intervention (PCF), and anticipated outcomes. Determinants and implementation strategies were informed by a study describing the development of the EOVs [32], while the clinical intervention (PCF) draws on earlier work by Lid and colleagues [10,33]. Specific mechanisms of impact were not hypothesised *a priori* but were explored inductively through qualitative analysis, consistent with the exploratory aims of this feasibility study. Implementation outcomes (feasibility, acceptability, and adoption) were assessed in this study, while potential service- and patient-level outcomes are presented as hypothesised effects that require evaluation in future research [34]. A refined logic model with empirically identified mechanisms is presented in the Results section (Figure 2). The implementation strategy (EOVs) and the clinical intervention (PCF) are described in more detail in the following sections. This study is reported in line with the consolidated criteria for reporting qualitative research [35] (COREQ; see Supplementary File 1).

## Implementation strategy: educational outreach visits (EOVs)

Four EOVs aimed to improve GPs’ ability to identify and address alcohol-related health problems through clinically relevant discussions with patients. This implementation strategy was systematically developed using the BCW and COM-B model [32]. The EOVs met Norwegian continuous medical education requirements for GPs and participants included GPs and support staff (in Norway primarily health secretaries and laboratory assistants). The visits were planned with one to two months between sessions, with a total duration of approximately six months including summer holidays. Originally, sessions were designed to be highly interactive, with physical attendance for presenters and participants, group discussions and role-play. After the first session in the two first clinics, the delivery mode had to be changed from in-person to virtual delivery due to the COVID-19 pandemic. The sessions ran from February 2020 to May 2021.

The agenda covered education in PCF, motivational interviewing, ethical considerations regarding health requirements for driver’s licenses, and interaction with addictive drugs (see Supplementary File 2 for a full agenda). To support behavioural repetition and habit formation, the sessions also included practical tools such as a list of clinically relevant conditions where PCF could be applied, and exercises where GPs developed specific action plans detailing when, where, and how they would use PCF in practice. Objective assessment tools, including the use of liver enzyme measurements, were also introduced as examples of data-driven approaches to support patient engagement and motivation.

## Clinical intervention: pragmatic case finding (PCF)

The clinical intervention (i.e. PCF) was operationalised in a shortlist of conditions and situations where alcohol is relevant to patients’ clinical condition [10]. This list was provided as a laminated sheet to keep on the desk as an aide-mémoire for GPs. The criteria for the conditions on the list were (1) sound evidence that alcohol may cause, precipitate or complicate the condition or situation, (2) the condition or situation is common in general practice, (3) the condition or situation is not *alcohol-specific*, and (4) reducing or cutting alcohol is likely to improve the condition or situation. Examples are depression, sleep problems, hypertension and atrial fibrillation (see Supplementary File 3 for the complete shortlist).

## Sampling and recruitment

A purposive sample of four GP practices in Stavanger and Oslo, including three large practices (7–10 GPs + interns) and one medium-sized (4–6 GPs + intern), were invited and agreed to participate. We purposively selected larger clinics to enable team-based delivery and diversity of GP experience. Practices were recruited *via* existing networks. We aimed to recruit practices with five or more physicians with a balance based on age and sex, and with both GP specialists and GPs in training. No prior relationship was established with participants before the study. Participants received Continuous Medical Education (CME) accreditation; no direct financial compensation was provided.

## Data collection

### Semi-structured group interviews

Semi-structured group interviews were conducted after the completion of the EOVs. The first interview was conducted by SP (Associate Professor, PhD, male), with subsequent group interviews conducted by ALMN (Research Associate, MSc, female) at participants’ workplaces. Both SP and ALMN are experienced in mixed-methods research. Interviews took place at GP practices between November 27, 2020, and December 1, 2021, and lasted 26 to 41 min. Using a theory-informed topic guide (COM-B), participants were interviewed in small groups to enable reflection and building on colleagues’ perspectives. The interviewer attended in person or *via* video link, and no non-participants were present (see Supplementary Files 4 for topic guide). Each participant was interviewed once; no repeat interviews were conducted.

### Determinants of implementation behaviour questionnaire (DIBQ)

The Determinants of Implementation Behaviour Questionnaire (DIBQ) [36] was administered at baseline (prior to the EOVs) and immediately following completion of the final outreach visit. It assessed intervention effects on 12 domains of implementation behaviour, including knowledge, intentions, and social/professional roles. The 46-question survey included a five-point Likert scale ranging from ‘strongly disagree’ to ‘strongly agree’. Four additional questions asked about professional practices related to addressing alcohol use, for example, ‘From memory, with how many patients on your last 5 clinical days did you yourself address alcohol consumption?’. We adapted the original version of the DIBQ to assess changes in addressing alcohol using a PCF approach. Following a published translation protocol, the adapted English version of the DIBQ was translated into Norwegian language using a forward-backward translation process carried out by independent translators [37]. Discrepancies between the original English version and the back translated English version were analysed by a behavioural scientist (SP). Changes were then integrated into the final English (Supplementary File 5) and Norwegian language version (See Supplementary File 6).

## Data management

All data were saved on the secure server for research data at Stavanger University Hospital, only accessible by the research team. Semi-structured group interviews were audio-recorded with participants’ consent and transcribed verbatim for analysis. Transcripts were not returned to participants for comment or correction. No field notes were made during or after the interviews. All data were carefully anonymised to prevent identification of either the individual participant or the participating study site. Qualitative software (NVivo 12) was used to support data management, analysis and documentation.

## Qualitative data analysis

Data were analysed using Braun and Clarke’s [38] six-step thematic analysis. Our approach was abductive: coding was primarily inductive but informed by the content of the EOVs and implementation theory (TDF/COM-B), consistent with qualitative content analysis guidance [39] and abductive theorising [40]. As this was a feasibility study with a fixed sample of participating clinics, data saturation was not sought.

## Quantitative data analysis

The statistical software R (version 4.3.2) was used for quantitative analysis of DIBQ data. Domain scores for each participant were created by taking their average score in each domain. Pre–post changes in DIBQ domains (Table 2) were analysed using paired one-sided t-tests to test whether scores were higher after compared to before the intervention. In addition, non-parametric Wilcoxon signed-rank tests were run as a sensitivity check, given the ordinal nature of Likert-type items, and showed consistent results. The four additional questions about professional practices related to addressing alcohol use (Table 3) were analysed descriptively only, as these were open-ended count data with skewed distributions and occasional extreme values, making significance testing unreliable in the context of this small feasibility sample.

## Results

### Sample

A total of 34 GPs and 22 support staff attended at least one session; 24 GPs and 14 support staff attended at least three sessions. Nineteen GPs participated in all four sessions and completed the DIBQ questionnaire before the first session and after the final session. Ten GPs took part across four group interviews (one in each practice). Three of the clinics were self-owned, and one by the municipality. The self-owned clinics opted for sessions after closing time, and the last clinic chose daytime sessions. Table 1 provides an overview of the participant characteristics.

**Table 1.** Participant characteristics of group interviews and surveys.

Characteristic	DIBQ both before and after (*n* = 19)	Post-intervention group interviews (*n* = 10)
**Years since graduation**		
< 1 year	0	0
1-3 years	0	1
4-10 years	6	3
> 10 years	13	6
**Professional role**		
GP	18	7
Locum GP / intern or GP in training	1	3
**Sex**		
Female	9	3
Male	10	7

## Qualitative results

### Question 1: How acceptable and feasible are EOVs for embedding PCF in practice?

An overview of all themes and additional illustrative quotes relating to the acceptability and feasibility of the training intervention can be found in Supplementary File 7.

#### Theme 1: Relevance and learning experience

Participants responded positively to the course content, especially the ‘*relatable clinical examples and evidence*’ [GP 1, Practice 4], which helped bridge theory with practice. Some suggested more varied, hands-on activities and clearer guidance for homework assignments to strengthen skills and accountability.

#### Theme 2: Engagement and delivery format

Maintaining engagement was sometimes challenging due to session length, timing, and lengthy intervals between visits. The shift to virtual delivery during COVID-19 received mixed feedback: some valued convenience, while others found the format less interactive and less effective for learning.

#### Theme 3: Facilitator rapport and support

Participants valued the expertise and relatability of the lead facilitator, a GP specialised in alcohol-related health issues, who ‘*was one of us*’ and understood ‘*the challenges we face*’ [GP 1, Practice 4]. Additional facilitators, including those with lived experience and expertise in motivational interviewing, were also appreciated for enriching the sessions.

### Question 2: How does embedding PCF affect general practitioners’ (GPs) approaches to discussing alcohol with patients?

An overview of themes and additional illustrative quotes related to changes in practitioners’ approach to addressing alcohol consumption can be found in Supplementary File 8. These qualitative findings were subsequently integrated into a refined logic model (Figure 2), which maps the empirically identified mechanisms onto the framework introduced in Figure 1 to illustrate how the EOVs may have supported implementation of PCF in practice.

#### Theme 1: Understanding alcohol’s health impacts

##### Broadening awareness

Participants discussed that education on PCF broadened their understanding of alcohol’s effects, moving beyond ‘*addiction and abuse in a more usual sense’* [GP 3, Practice 3]. One GP described a greater awareness of how alcohol can exacerbate conditions like immune dysfunction, diabetes, high blood pressure, and mental health issues. Another GP applied this knowledge from EOVs to treat sleep disorders by addressing alcohol use and made discussions about alcohol a key preventive measure for patients with chronic conditions like diabetes and hypertension.

##### Managing chronic diseases

Participants recognised that PCF made discussions about alcohol a key preventive measure for patients with chronic conditions and high-risk factors like diabetes, hypertension, and being overweight. One GP noted that discussing alcohol reduction could lessen medication dependence and improve health outcomes in patients with chronic conditions.

#### Theme 2: Recognising hidden cases

##### Sustaining awareness of hidden cases

GPs reported that the EOVs helped them to ‘*keep alcohol-related health issues warm in our minds*’ [GP 1, Practice 2], preventing attention from drifting back to only the most severe cases. They became more alert to patients whose alcohol use was less obvious but still clinically relevant, noting that such patients might be especially receptive to change given their otherwise good functioning.

##### Using tools and monitoring

Concrete tools were seen as particularly valuable in identifying and addressing hidden cases. Liver enzyme measurements and other markers made alcohol’s health effects visible, while observing improvements such as weight loss or lower blood pressure provided GPs with “*a boost of motivation*” [GP 2, Practice 4] to discuss alcohol more proactively.

#### Theme 3. Normalising alcohol discussions

##### Reducing stigma and focusing on moderation

Participants explained that PCF supported non-judgmental, patient-centred conversations that avoided labels such as ‘*alcoholic*’ and integrated alcohol into broader health discussions. This approach helped patients feel more at ease and encouraged openness. GPs also found that emphasising reduction rather than abstinence created less pressure and supported realistic, achievable goals for change.

##### Building communication skills

Motivational interviewing resources, including expert videos, were perceived as valuable for developing new language and strategies. These techniques enabled GPs to raise alcohol more confidently and effectively, without alienating patients.

#### Theme 4: Changing clinical habits

##### Balancing motivation and challenges

GPs reported increased motivation to raise alcohol more regularly, shifting from a lecturing style to a more holistic, health-oriented approach. At the same time, they acknowledged the difficulty of changing long-standing routines, describing progress as ‘*a slow process*’ [GP 5, Practice 1].

##### Seizing routine opportunities

Participants identified natural opportunities to address alcohol in everyday consultations. For example, one GP noted that advising patients to reduce consumption before surgery could enhance outcomes: ‘*you can remind people that it is important to reduce your consumption before a surgery, so the results of the surgery are much better*’ [GP 1, Practice 3]. The EOVs provided inspiration for such opportunities, reinforcing the idea that alcohol conversations could be integrated into routine care.

## Quantitative results

### Question 3: How did determinants of implementation behaviour change from before to after the EOVs?

**Figure 3.**
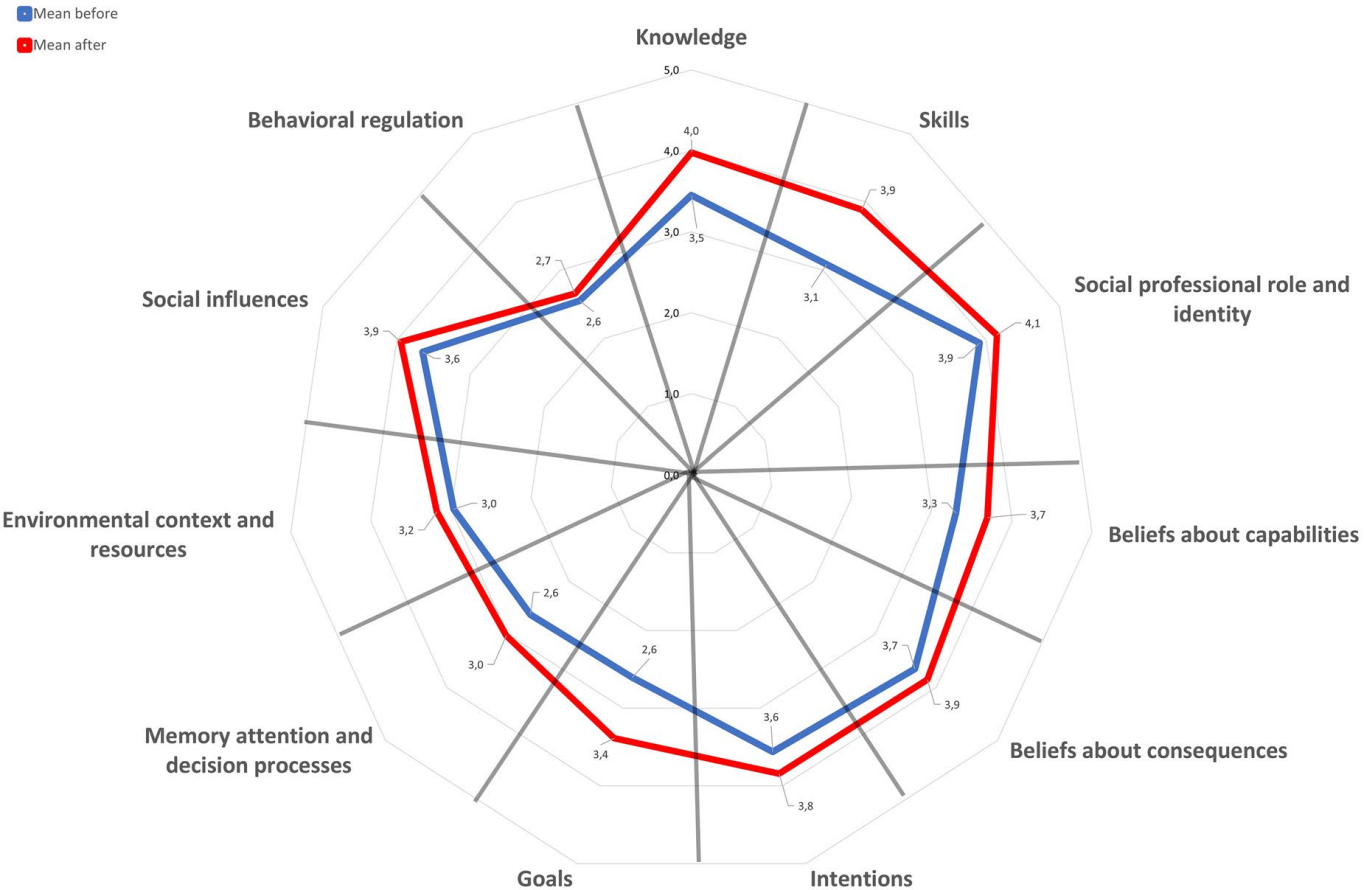
Changes across Determinants of Implementation Behaviour Questionnaire (DIBQ) domains from before compared to after the EOVs.

A total of 19 GPs completed the DIBQ before and after the EOVs. Significant increases (*p* < 0.05) were observed in eight of the eleven domains, including *knowledge*, *skills*, *social professional role and identity*, *beliefs about capabilities*, *beliefs about consequences*, *goals, memory attention and decision processes,* and *social influences*, indicating a meaningful impact from the intervention (see Table 2). No significant changes were observed in three of the eleven domains, including *intentions, environmental context and resources*, and *behavioural regulation*, suggesting less effectiveness in these domains (see Table 2). Figure 3 displays a petal chart of pre–post differences across DIBQ domains, highlighting the domains in which GPs reported the greatest improvements after the EOVs.

**Table 2.** Determinants of implementation behaviour domain scores (before/after), cronbach’s alphas, and significance values.

Scale	Number of domain items	Scale reliability (α)	Mean (SD) before	Mean (SD) after	*p* values for one-sided paired t-tests comparing after with before
Knowledge	5	0.7	3.5 (0.4)	4.0 (0.3)	*p* < 0.01
Skills	3	0.8	3.1 (0.6)	3.9 (0.5)	*p* < 0.01
Social professional role and identity	3	0.4	3.9 (0.4)	4.1 (0.6)	*p* = 0.03
Beliefs about capabilities	6	0.8	3.3 (0.6)	3.7 (0.5)	*p* = 0.02
Beliefs about consequences	5	0.5	3.7 (0.3)	3.9 (0.4)	*p* = 0.047
Intentions	3	0.6	3.6 (0.7)	3.8 (0.6)	*p* = 0.051
Goals	3	0.8	2.6 (0.4)	3.4 (0.5)	*p* < 0.01
Memory attention and decision processes	2	0.2	2.6 (0.6)	3.0 (0.6)	*p* = 0.02
Environmental context and resources	3	0.4	3.0 (0.6)	3.2 (0.5)	*p* = 0.09
Social influences	3	0.7	3.6 (0.5)	3.9 (0.5)	*p* < 0.01
Behavioural regulation	4	0.9	2.6 (0.8)	2.7 (0.7)	*p* = 0.25

**Table 3.** Professional practice of addressing alcohol before and after the educational outreach visits.

Questions about professional practice	Alcohol conversations before Mean (SD) *N* = 19	Alcohol conversations after Mean (SD) *N* = 19
1. How many patient consultations do you normally have during a clinical day?	19.3 (4.7)	19.6 (6.3)
2. From memory, with how many patients on your last 5 clinical days did you yourself address alcohol consumption?	2.2 (2.0)	3.1 (4.5)
3. From memory, for how many patients on your last 5 clinical days did you offer advice or help related to alcohol?	0.4 (0.7)	1.4 (4.5)
4. From memory, how many patients on your last 5 clinical days asked you themselves about anything alcohol related?	1.0 (1.4)	1.8 (1.9)
5. From memory, how many relatives of patients from the last month reached out to you about the patient and his/her alcohol use?	0.1 (0.3)	0.3 (0.5)

Table 3 presents the DIBQ questions about GPs’ professional practice of addressing alcohol before and after the EOVs. On average, GPs reported having around 19 consultations during a clinical day. There was an increase in the average number of patients they addressed alcohol with in the last five clinical days (from 2.2 to 3.1). There was also an increase in the average number of patients they offered alcohol related advice to in the last five clinical days (from 0.1 to 1.4) and in the average number of patients (from 1 to 1.8) and relatives (from 0.1 to 0.3) who asked the GPs alcohol related questions in the last five clinical days.

We calculated internal reliability scores using Cronbach’s alpha (see Table 2). Six domains demonstrated good reliability, while five fell below the acceptable threshold of 0.7, indicating potential issues with item consistency or construct validity. Low scores may signify a lack of coherence among items or excessive variance.

## Discussion

### Summary of findings

This mixed methods feasibility study evaluated educational outreach visits (EOVs) as an implementation strategy to embed pragmatic case finding (PCF) in Norwegian general practice. The EOVs were feasible and acceptable and supported greater general practitioner (GP) engagement with alcohol-related health issues. Qualitative analysis identified four mechanisms: broadening awareness of alcohol’s health impacts, recognising hidden cases, normalising discussions, and adapting clinical habits. Practitioners valued the clinical relevance of the content but highlighted the need for more interactive activities to build skills such as motivational interviewing. Quantitative findings showed modest but positive changes in confidence, skills, and frequency of addressing alcohol, consistent with the qualitative themes. Together, these findings suggest that EOVs can support the implementation of PCF and promote a more holistic, patient-centred approach to alcohol in primary care.

## Strengths and limitations

A key strength of this study was its theory-based approach to tailoring the EOVs to GPs’ needs, incorporating practical tools like liver enzyme measurements, and scheduling sessions in their practices free of charge. The use of both qualitative and quantitative methods provided a comprehensive understanding of the implementation strategy and its influence on professional practice. However, limitations include the small sample size, restricted to four GP clinics in Stavanger and Oslo, which may limit the generalisability of the findings. Self-reported practice measures may be susceptible to social desirability bias; this should be considered when interpreting changes reported in Table 3. In addition, we did not formally assess the fidelity of the EOVs or the PCF approach, which limits our ability to determine whether all components were delivered and received as intended. Lastly, the COVID-19 pandemic necessitated a shift to online learning, which posed challenges for participant engagement and continuous learning.

## Comparisons with existing literature

The findings align with existing literature on continuing medical education, which emphasises that interactive and spaced learning sessions enhance knowledge retention and motivation to apply new skills in practice [41]. The expanded understanding of alcohol’s role beyond addiction, incorporating it into behaviour change and preventive care strategies for conditions such as hypertension and diabetes, echoes recent studies highlighting the importance of holistic care approaches [42,43]. This holistic approach fosters a non-judgemental discussion of alcohol, potentially reducing stigma. Recent studies on barriers to treatment seeking for alcohol use disorders (AUD) in Denmark found that the barriers increased with increased severity of AUD, and that both higher and lower stigma was associated with a preference for consulting a GP rather than other treatment options [9,44]. The identified synergistic risks between alcohol and other factors, such as smoking or excess weight, support the notion that addressing alcohol consumption in primary care can yield compounded health benefits [45]. This is consistent with harm-reduction strategies and aligns with studies suggesting that incremental changes in alcohol consumption, rather than focusing solely on abstinence, can significantly improve health outcomes [46].

Quantitative findings revealed modest yet positive changes in professional practice and attitudes. In line with theories of habit, these small improvements across determinants of implementation behaviour, highlight the importance of consistent reinforcement and practice to support sustained behaviour change [47]. In the EOVs, we incorporated tools and techniques to support behavioural repetition and habit formation, including a list of clinically relevant conditions [48]. GPs were encouraged to create specific ‘action plans’ detailing when, where, and how they would implement PCF [32,49]. To further facilitate habit change, future training could include post-intervention support mechanisms, such as reminders or peer discussion groups, which would reinforce learned behaviour over time. Additionally, embedding alcohol conversations into routine situations, such as health checks, may help sustain this new approach, making it a regular part of GPs’ practice [10]. Yet clinical habits are also shaped by broader cultural norms, stigma, and biases around alcohol, which may discourage discussion even when clinically relevant. Training strategies should therefore combine habit-building techniques with approaches that normalise alcohol conversations within routine care [47].

The use of practical tools, such as liver enzyme measurements, facilitated more objective, data-driven conversations. This approach enabled GPs and patients to understand alcohol’s impact more concretely, supporting motivation for behaviour change. Recently, phosphatidyl ethanol (PEth), an alcohol-specific lab test, has become more commonly used in Sweden for routine follow-up of hypertension [50]. In Norway, PEth has been tested in population studies and hospital settings and is now commonly used in primary care primarily in relation to alcohol use disorders [51,52]. The value that participants placed on objective monitoring also suggests that incorporating structured follow-up assessments could help maintain these skills and sustain proactive identification of alcohol-related health problems.

The IRLM [31] provided a useful framework for linking this feasibility study with earlier work on PCF [10,33] and educational outreach [32]. In keeping with the study’s exploratory purpose, the initial logic model served as a planning framework, while the refined version presented in the Results section synthesises inductively identified mechanisms. This approach was hypothesis-generating rather than confirmatory, providing preliminary insights into potential causal mechanisms that warrant testing in future evaluation (e.g. mediation or realist analyses). Using the IRLM in this reflective way supports theory-building and helps consolidate emerging evidence across implementation contexts.

## Implications for research and practice

Future studies should be developed together with both patients and clinicians in primary care, and focus on strategies applicable in everyday clinical practice [20]. As drinking alcohol is a normalised activity in western culture and alcohol consumption can be relevant for many common health problems in primary care, we need to normalise addressing alcohol in diagnostic work, treatment and follow-up for these health problems. Screening all patients for signs of risky or harmful alcohol consumption is not viable, but we need more research on pragmatic strategies, for example, PCF or targeted screening, in large samples and in different cultures to establish whether such strategies are sustainable and effective, in terms of reduced alcohol consumption and improvement in health outcomes. However, there is sufficient evidence for including such strategies in clinical practice without waiting for more evidence.

## Conclusion

There is a growing interest in clinically relevant strategies for alcohol SBI in primary care. The PCF approach provides a systematic way of addressing alcohol in relation to common health problems and aligns with existing brief intervention approaches. This feasibility study demonstrates that tailored, theory-based EOVs were a feasible and acceptable strategy to support implementation of PCF in Norwegian primary care, helping to build GP capability and confidence in applying clinical judgement. Preliminary signals suggested practitioner-level change, including greater awareness of alcohol’s health impacts, improved recognition of hidden cases, and more routine discussions of alcohol, although challenges in changing established clinical habits remained. These findings support educational outreach as a promising strategy for embedding PCF in practice and provide guidance for future research and implementation.

## Supplementary Material

PCF feasibility_supplementary files_revised_SJPHC_v2.docx

## Data Availability

The datasets generated and/or analysed during the current study are not publicly available because this would likely compromise participants’ anonymity. Some descriptive data may be available from the corresponding author on reasonable request.
