# Supplementary material for: Implementing pragmatic case finding to address alcohol use in general practice: a mixed methods feasibility study
Source: Scand J Prim Health Care. 2025 Dec 19;44(1):2598835. doi: 10.1080/02813432.2025.2598835 (PMC12720610; doi:10.1080/02813432.2025.2598835)
Supplement: PCF feasibility_supplementary files_revised_SJPHC_v2.docx [file IPRI_A_2598835_SM4127.docx]

***Supplementary Files***

**Supplementary file 1.** Consolidated criteria for reporting qualitative studies (COREQ): 32-item checklist

Developed from:

Tong A, Sainsbury P, Craig J. Consolidated criteria for reporting qualitative research (COREQ): a 32-item checklist for interviews and focus groups. *International Journal for Quality in Health Care*. 2007. Volume 19, Number 6: pp. 349 – 357

| **No. Item** | **Guide questions/description** | **Reported on Page #** |
| --- | --- | --- |
| **Domain 1: Research team and reﬂexivity** |  |  |
| *Personal Characteristics* |  |  |
| 1. Interviewer/facilitator | Which author/s conducted the interview or focus group? | 6 (Methods: Data collection) |
| 2. Credentials | What were the researcher’s credentials? E.g. PhD, MD | 6 (Methods: Research team) |
| 3. Occupation | What was their occupation at the time of the study? | 6 (Methods: Research team) |
| 4. Gender | Was the researcher male or female? | 6 (Methods: Research team) |
| 5. Experience and training | What experience or training did the researcher have? | 6 (Methods: Research team) |
| *Relationship with participants* |  |  |
| 6. Relationship established | Was a relationship established prior to study commencement? | 6 (Methods: Sampling and recruitment) |
| 7. Participant knowledge of the interviewer | What did the participants know about the researcher? e.g. personal goals, reasons for doing the research | 6 (Methods: Sampling and recruitment) |
| 8. Interviewer characteristics | What characteristics were reported about the interviewer/facilitator? e.g. Bias, assumptions, reasons and interests in the research topic | 6 (Methods: Semi-structured group interviews) |
| **Domain 2: study design** |  |  |
| *Theoretical framework* |  |  |
| 9. Methodological orientation and Theory | What methodological orientation was stated to underpin the study? e.g. grounded theory, discourse analysis, ethnography, phenomenology, content analysis | 5–6 (Methods: Qualitative analysis) |
| *Participant selection* |  |  |
| 10. Sampling | How were participants selected? e.g. purposive, convenience, consecutive, snowball | 6 (Methods: Participants and recruitment) |
| 11. Method of approach | How were participants approached? e.g. face-to-face, telephone, mail, email | 6 (Methods: Participants and recruitment) |
| 12. Sample size | How many participants were in the study? | 7 (Results: Participants) |
| 13. Non-participation | How many people refused to participate or dropped out? Reasons? | 7 (Results: Participants) |
| *Setting* |  |  |
| 14. Setting of data collection | Where was the data collected? e.g. home, clinic, workplace | 6 (Methods: Data collection) |
| 15. Presence of non-participants | Was anyone else present besides the participants and researchers? | 6 (Methods: Semi-structured group interviews) |
| 16. Description of sample | What are the important characteristics of the sample? e.g. demographic data | 7 (Results: Participants) |
| *Data collection* |  |  |
| 17. Interview guide | Were questions, prompts, guides provided by the authors? Was it pilot tested? | 6 (Methods: Data collection) |
| 18. Repeat interviews | Were repeat interviews carried out? If yes, how many? | 6 (Methods: Data collection) |
| 19. Audio/visual recording | Did the research use audio or visual recording to collect the data? | 6 (Methods: Data collection) |
| 20. Field notes | Were ﬁeld notes made during and/or after the interview or focus group? | 7 (Methods: Data collection) |
| 21. Duration | What was the duration of the inter views or focus group? | 6 (Methods: Data collection) |
| 22. Data saturation | Was data saturation discussed? | 7 (Methods: Data collection) |
| 23. Transcripts returned | Were transcripts returned to participants for comment and/or correction? | 7 (Methods: Data collection) |
| **Domain 3: analysis and ﬁndings** |  |  |
| *Data analysis* |  |  |
| 24. Number of data coders | How many data coders coded the data? | 6 (Methods: Qualitative analysis) |
| 25. Description of the coding tree | Did authors provide a description of the coding tree? | 11 (Results: Themes and subthemes described) |
| 26. Derivation of themes | Were themes identiﬁed in advance or derived from the data? | 6 (Methods: Qualitative analysis – abductive) |
| 27. Software | What software, if applicable, was used to manage the data? | 6 (Methods: Qualitative analysis) |
| 28. Participant checking | Did participants provide feedback on the ﬁndings? | n/a |
| *Reporting* |  |  |
| 29. Quotations presented | Were participant quotations presented to illustrate the themes/ﬁndings? Was each quotation identiﬁed? e.g. participant number | 8–11 (Results: Themes with quotes) |
| 30. Data and ﬁndings consistent | Was there consistency between the data presented and the ﬁndings? | 8–11 (Results: Themes with quotes) |
| 31. Clarity of major themes | Were major themes clearly presented in the ﬁndings? | 8–11 (Results: Four themes reported) |
| 32. Clarity of minor themes | Is there a description of diverse cases or discussion of minor themes? | 8–11 (Results: Subthemes reported) |

**Supplementary file 2.** Overview of educational outreach visit agenda

Alcohol-related health problems in general practice

four EDUCATIONAL OUTREACH VISITS (eovS) with home assignments

ADD LOCATION AND DATES

EOV 1 (3x45 min), Doctors and employees

ADD FACILITATOR NAMES

- Welcome and information about the OUTREACH VISIT.
- Introduction to *Endre*, web-based adjustment support for patients. Discussion of use of Endre in own practice.
- Relationship between alcohol consumption, health and illness in general practice. Pragmatic case finding. Conditions where alcohol has an impact. Addictive prescription drugs. Barriers when taking about alcohol. What does patients think of being asked about alcohol?
- Introduction into ageing and alcohol consumption.
- Home assignment – conditions where alcohol has an impact.
- Summary and comments.

EOV 2 (4x45 min) – Doctors and employees

ADD FACILITATOR NAMES

- *Endre* – experiences and frustrations.
- Alcohol – easier than you think (3x45 min). Short on experiences with the home assignment. Different health effects of alcohol. Practical strategies for the GP. Clinical examples and evidence. GP`s toolbox. White periods, “halving test”. Ageing and alcohol.
- Brief group discussion on how to start a dialogue on alcohol with your patient.
- Home assignment for next time – strategies to prepare and start conversations on alcohol.
- Summary and comments.

EOV 3 (4x45 min) – DOCTORS AND EMPLOYEES

ADD FACILITATOR NAMES

- *Endre* - experiences and frustrations.
- Alcohol – easier than you think (2x45 min). Short on experiences with the home assignment. Follow-up on previous EOV.
- Motivation and change (60min) with practical exercises.
- Home assignment for next time – tools from your toolbox.
- Summary and comments.

EOV 4 (4x45 min) – doctors and employees

ADD FACILITATOR NAMES

- *Endre* – experience and further use – is it a useful tool?
- Brief discussion on home assignment, reflections and sharing of experiences.
- Motivation and change (45min) – part 2.
- Ethical and practical problems related to driver`s license (45min).
- Short on addictive prescription drugs and alcohol (30min).
- Collaboration and referral to other actors in the municipal and specialized health services (30 min).
- Evaluation.

**Supplementary file 3.** Shortlist of conditions and situations where alcohol is relevant to the patient’s clinical condition

**Conditions and symptoms**

Mental health Depression

Anxiety

Sleep disorders

Age-related reduction of cognitive function

Gastrointestinal Dyspepsia

Diarrhea

Cardiovascular Hypertension

Arrythmia

Ortopedic Knee and hip replacement – surgical risks

Dermathology Exacerbation of psoriasis

Seborrhoic dermatitis

Chronic conditions Annual check-up, e.g. diabetes, heart disease, chronic inflammatory disorders

Miscellaneous Vertigo, dizziness, wobbliness, falling

Chronic pain

High normal or elevated liver enzymes, especially GGT

Age related physical deteriorations

**Contexts**

Life crises Work-related problems

Family problems

Losing next of kin

Major life changes Retirement

Medications Use of addictive drugs

Obesity

Trauma

Smoking cessation

**Supplementary file 4.** Interview topic guide

**Introduction**

- Introduce yourself
- Introduce the purpose of the interview
  - To understand their views on the educational outreach visits (EOVs) on alcohol and related health problems
- Inform about: Audio recording, anonymisation, stopping interview
- Reaffirm participant consent

**Background information (to be collected prior to the interview)**

- What is your role in the group practice? (will collect this data ahead of interview)
- How long have you been working as a GP? (will collect this data ahead of interview)

**General questions**

- What was your overall impression of the clinical topic EOVs?
  - Any likes / dislikes
- What did you think of the format of delivery?
  - Number / duration of the sessions
  - Group format including all members of staff
  - Virtual delivery of certain components
  - Homework assignments
- Was there anything that facilitated or hindered your learning experience?

**Pragmatic case finding questions**

- How relevant was the topic (alcohol) for your own practice?
- To what extent did the EOVs help you think about alcohol-related health problems in a different way?
- Did you learn more about the different medical conditions in which alcohol may be relevant?
- To what extent do you feel you can use this new knowledge to support your patients with changing their alcohol habits / helping the patient improve their health?

**COM-B questions**

- Do you feel like you have the necessary time and resources to apply the new knowledge / skills in your own practice? If not, are there certain elements of the training that you will use?
- How motivated are you to apply the knowledge / skills that you have learned in your own practice?
  - Anything that makes you less / more motivated?
- Have the EOVs helped you develop a habit around addressing alcohol with your patients?
  - How long did it take you to form a routine around addressing alcohol?
  - If so, what helped making it a habit / what were the barriers to making it routine?
- How often do you consult patients who suffer from alcohol-related health problems?
  - Has there been a change in the number patients who you consult about alcohol?

**Final remarks**

- Any final comments?
- Thank you for your participation

**Supplementary File 5.** English version of adapted Determinants of Implementation Behaviour Questionnaire (DIBQ)

ALCOHOL HABITS - PATIENT CONSULTATIONS IN GENERAL MEDICAL PRACTICE

WHO IS THIS QUESTIONNAIRE FOR?

*The questionnaire is for all GP’s in the practice who are taking part in the course ‘Alcohol Related Health Problems in General Practice’.*

WHAT IS THIS QUESTIONNAIRE ABOUT?

The questionnaire will give us a greater understanding of how GP’s address alcohol related topics with their patients.

HOW LONG WILL IT TAKE?

It will take **10-12 minutes** to complete the questionnaire.

HOW MANY TIMES WILL I HAVE TO COMPLETE THIS QUESTIONNAIRE?

You will have to fill it out twice. The second time will be in about six months, after the last part of the course. This is to identify possible changes in your points of view after completing the course.

CONFIDENTIALITY

All answers are confidential, and all results will be presented in a way that does not identify individual participants.

Thank you for taking part in this project!


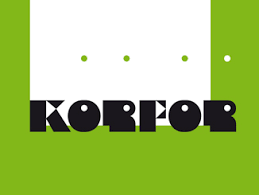


Your background

I AM A MAN WOMAN

MY AGE

GRADUATION YEAR

TOTAL HOURS OF DIRECT PATIENT CONTACT PER WEEK

I AM A GP LOCUM intern

| Now you can complete the rest of the questionnaire. Your immediate response to the questions is often the most accurate. Some of the questions are very similar. This is because we want to investigate a particular topic in different ways.   - It is important for the study that you answer **all** of the questions. Please check over the form when you get to the end to see if you have missed any questions. - Read the text in the green box at the top of each page. The text is the same on every page and is there to remind you of the context for answering the questions. - Answer the questions by drawing a circle around your chosen number. - **Do you have any questions before you start? Please see the contact information at the end of the questionnaire.** |
| --- |
|  |

a. QUESTIONS ABOUT YOUR KNOWLEDGE

|  | **Disagree strongly** | **Disagree** | **Neither agree nor disagree** | **Agree** | **Agree Strongly** |
| --- | --- | --- | --- | --- | --- |
| 1. I know **when** it is the right time to ask about alcohol habits during a consultation. |  |  |  |  |  |
| 2. I know **how** to ask about alcohol habits during a consultation. |  |  |  |  |  |
| 3. I am aware of **effective initiatives** for reducing alcohol use, that I can offer to **my patients** |  |  |  |  |  |
| 4. I am familiar with the **foundation of expertise** for initiatives to deal with alcohol problems in general practice. |  |  |  |  |  |
| 5. I understand how **alcohol use can affect,** and complicate, many health problems (For example, mental health problems and many chronic somatic conditions). |  |  |  |  |  |

b. QUESTIONS ABOUT YOUR MEDICAL PRACTICE

| 1. How many consultations do you have during a normal working day? |  |
| --- | --- |
| 2. From memory, how many patients during the **last five working days** **did you** ask about alcohol use? |  |
| 3. From memory, how many patients during the **last five working days asked you** about any alcohol related issues? |  |
| 4. From memory, how many patients during the **last five working days did you** give advice or help related to alcohol habits? |  |
| 5. From memory, how many **family members of patients** have contacted you about a patient’s alcohol use **during the last month**? |  |

*Answer using a number.*

| **Answer the following questions in the context that you** are either:  **Taking the initiative** to talk about alcohol use **as a part of a routine consultation;** or,  Asking about alcohol habits because the patient **has possible alcohol related health problems.** |
| --- |

|  | **Disagree strongly** | **Disagree** | **Neither agree nor disagree** | **Agree** | **Agree Strongly** |
| --- | --- | --- | --- | --- | --- |
| C1. I have had **adequate training** in asking about alcohol habits and offering follow-up during consultations. |  |  |  |  |  |
| C2. I have the **necessary skills** to ask about alcohol habits and offer follow-up during consultations. |  |  |  |  |  |
| C3. I feel **professionally secure** when I ask about alcohol habits and offer follow-up during consultations. |  |  |  |  |  |
| D1. To ask about alcohol habits and offer follow-up is  **a part of my every-day work** as a GP. |  |  |  |  |  |
| D2. It is my **responsibility** as a GP to ask about alcohol habits and offer follow-up. |  |  |  |  |  |
| D3. To ask about alcohol habits and offer follow-up **is a natural part** of my role as a GP. |  |  |  |  |  |

| **Answer the following questions in the context that you** are either:  **Taking the initiative** to talk about alcohol use **as a part of a routine consultation;** or,  Asking about alcohol habits because the patient **has possible alcohol related health problems.** | | | | | | | | | | |  |
| --- | --- | --- | --- | --- | --- | --- | --- | --- | --- | --- | --- |
| **I fell confident that I can ask about alcohol habits and offer follow-up during consultations, even if…** | | **Disagree strongly** | | **Disagree** | | **Neither agree nor disagree** | | **Agree** | | **Agree Strongly** | |
| E1. …the patient is **not motivated** | |  | |  | |  | |  | |  | |
| E2. …the patient thinks it is **stigmatising them.** | |  | |  | |  | |  | |  | |
| E3. …it can be **uncomfortable** for me. | |  | |  | |  | |  | |  | |
| E4. …it will lead to **more work** for me. | |  | |  | |  | |  | |  | |
| E5. …it can be **embarrassing for the patient, or seem offensive.** | |  | |  | |  | |  | |  | |
| E6. …**I am worried that** the patient may not meet the health related criteria required for a drivers license. | |  | |  | |  | |  | |  | |
| **If I ask about alcohol habits and offer follow-up consultations, it will…** | **Disagree strongly** | | **Disagree** | | **Neither agree nor disagree** | | **Agree** | | **Agree Strongly** | |  |
| F1. …**be good** for the patients health. |  | |  | |  | |  | |  | |  |
| F2. … **be negative for the doctor -patient relationship.** |  | |  | |  | |  | |  | |  |
| F3**. … suppress the issue the patient actually wanted** to bring up. |  | |  | |  | |  | |  | |  |
| F4 … be especially useful for my **elderly patients.** |  | |  | |  | |  | |  | |  |
| F5. … be especially useful for my patients with **mental health issues.** |  | |  | |  | |  | |  | |  |

| **Answer the following questions in the context that you** are either:  **Taking the initiative** to talk about alcohol use **as a part of a routine consultation;** or,  Asking about alcohol habits because the patient **has possible alcohol related health problems.** | | | | | | | | | | |  |
| --- | --- | --- | --- | --- | --- | --- | --- | --- | --- | --- | --- |
|  | | **Disagree strongly** | | **Disagree** | | **Neither agree nor disagree** | | **Agree** | | **Agree Strongly** | |
| G1. **I am committed** to asking about alcohol habits and to offer follow-up when it is relevant. | |  | |  | |  | |  | |  | |
| G2. **I am planning to ask** about alcohol habits and offer follow-up when it is relevant. | |  | |  | |  | |  | |  | |
| G3. **I will ask** all my patients **with mental health problems, or other chronic conditions,** about their alcohol habits | |  | |  | |  | |  | |  | |
| H1. I have a clear plan about **how** I will ask about alcohol habits and offer follow-up. | |  | |  | |  | |  | |  | |
| H2. I have a clear plan for **in which situations** I will ask about alcohol habits and offer follow-up. | |  | |  | |  | |  | |  | |
| H3. I have a clear plan about **how often** I will ask about alcohol habits and offer follow-up. | |  | |  | |  | |  | |  | |
| I1. In the last month **I have often forgotten to** ask patients about their alcohol habits where this would have been relevant. | |  | |  | |  | |  | |  | |
| I2. When I have to **focus** on asking about alcohol habits, I am often **less attentive to the patient’s own agenda.** | |  | |  | |  | |  | |  | |
| J1. I **have enough time and resources to** ask about alcohol habits and offer follow-up. | |  | |  | |  | |  | |  | |
| J2. I **can source the relevant help** for my patients with the **local council** if they need it. | |  | |  | |  | |  | |  | |
| J3. I **can source the relevant help** for my patients with **specialist health services** if they need it. | |  | |  | |  | |  | |  | |
| **Answer the following questions in the context that you** are either:  **Taking the initiative** to talk about alcohol use **as a part of a routine consultation;** or,  Asking about alcohol habits because the patient **has possible alcohol related health problems.** | | | | | | | | | | |  |
|  | **Disagree strongly** | | **Disagree** | | **Neither agree nor disagree** | | **Agree** | | **Agree Strongly** | | |
| K1. **My co-workers in the practice support** my work by asking about alcohol habits and offering follow-up. |  | |  | |  | |  | |  | | |
| K2. **Most people in my professional network** (experienced colleagues, advisors, members of my group of colleagues/advisory group) **support** the initiative to ask about alcohol habits and offer follow-up. |  | |  | |  | |  | |  | | |
| K3. **Positive attitudes amongst members of my practice encourages me** to ask about alcohol habits and offer follow-up. |  | |  | |  | |  | |  | | |

| **Asking about alcohol habits during consultations is something I …** | **Disagree strongly** | **Disagree** | **Neither agree nor disagree** | **Agree** | **Agree Strongly** |
| --- | --- | --- | --- | --- | --- |
| L1. … do **automatically.** |  |  |  |  |  |
| L2. … do **without having to remind myself.** |  |  |  |  |  |
| L3. … do **without thinking about.** |  |  |  |  |  |
| L4. … start to do **before I am conscious that** I am doing it. |  |  |  |  |  |

A = Knowledge
B = Medical Practice
C = Skills
D = Social/professional role and identify (identity?)
E = Beliefs about capabilities
F = Beliefs about consequences
G = Intentions
H = Goals
I = Memory, attention and decision processes
J = Enviromental context and resources
K = Social influences
L = Behavioural regulation

THANK YOU,

YOU HAVE NOW COMPLETED THE QUESTIONNAIRE!

DO YOU HAVE ANY QUESTIONS OR COMMENTS?

IF YOU HAVE QUESTIONS OR COMMENTS ABOUT THE STUDY, PLEASE CONTACT:

Researcher details

**Supplementary file 6.** Norwegian version of adapted Determinants of Implementation Behaviour Questionnaire (DIBQ)

Samtaler med pasienter om alkoholvaner i allmennpraksis

Hvem skal svare på spørreskjemaet?

Spørreskjemaet er for alle legene på legesenteret som deltar på emnekurset *alkoholrelaterte helseproblemer i allmennpraksis*

Hva handler spørreskjemaet om?

Spørreskjemaet skal gi oss større forståelse av hvordan du som allmennlege tar opp alkoholrelaterte tema med sine pasienter.

Hvor lang tid tar det?

Utprøving har vist at de fleste bruker **10-12 minutter** på å fullføre skjemaet.

Hvor mange ganger må jeg fylle ut skjemaet?

Dette skjemaet skal du fylle ut to ganger**.** Du skal fylle ut skjemaet for andre gang om ca. seks måneder, etter siste møte i kursrekken. Dette er for å kunne identifisere mulige endringer i synspunkter etter gjennomført kursrekke.

Konfidensialitet

Alle svar er konfidensielle, og alle resultat vil bli presentert på måter som ikke identifiserer deltakerne.

Tusen takk for at du deltar i dette prosjektet!


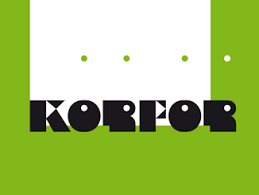


Din bakgrunn

Jeg er Mann Kvinne

Alder

uteksaminert i år

Antall timer med direkte pasientkontakt i uken

Jeg er fastlege ALis/vikar lis1

| Nå kan du fullføre resten av spørreskjemaet. Ditt umiddelbare svar på spørsmålene er ofte det mest riktige. Noen spørsmål kan ligne på hverandre. Det er fordi vi vil undersøke et tema på ulike måter.   - Det er viktig for studien at du besvarer **alle** spørsmålene. Se gjerne over at du **ikke har glemt noen spørsmål**. - Les den **grønne boksen på toppen av hver side**. Den er identisk på alle sidene fra side 4, og den skal minne deg på sammenhengen når du svarer på spørsmålene. - Svar på spørsmålene ved å tegne en **sirkel** rundt valgt tall. - **Lurer du på noe før du begynner? Se kontakt-info til slutt i skjemaet.** |
| --- |

1. spørsmål om Din kunnskap

|  | Helt uenig | Uenig | Hverken enig/uenig | Enig | Helt enig |
| --- | --- | --- | --- | --- | --- |
| 1. Jeg vet **når** det er aktuelt å spørre om alkoholvaner i konsultasjoner | 1 | 2 | 3 | 4 | 5 |
| 1. Jeg vet **hvordan** jeg skal spørre om alkoholvaner i konsultasjoner | 1 | 2 | 3 | 4 | 5 |
| 1. Jeg kjenner til **effektive tiltak** for å redusere alkoholforbruket, som jeg kan tilby **mine pasienter** | 1 | 2 | 3 | 4 | 5 |
| 1. Jeg er kjent med **kunnskapsgrunnlaget** for tiltak mot alkoholproblemer i allmennpraksis | 1 | 2 | 3 | 4 | 5 |
| 1. Jeg er klar over hvordan **alkoholforbruk kan påvirke** og komplisere mange helseproblem (f.eks. psykiske lidelser, mange kroniske somatiske tilstander) | 1 | 2 | 3 | 4 | 5 |

1. spørsmål om Din legepraksis

| 1. Hvor mange konsultasjoner., inkludert e-konsultasjoner, har du i løpet av en vanlig arbeidsdag? |  |
| --- | --- |
| 1. Fra hukommelsen, hvor mange pasienter i løpet av de fem siste praksisdagene spurte du på eget initiativ om alkoholforbruk? |  |
| 1. Fra hukommelsen, hvor mange pasienter i løpet av de fem siste praksisdagene spurte deg om noe som har med alkohol å gjøre? |  |
| 1. Fra hukommelsen, hvor mange pasienter i løpet av de fem siste praksisdagene ga du råd eller hjelp knyttet til alkoholvaner? |  |
| 1. Fra hukommelsen, hvor mange familiemedlemmer eller andre nærstående i løpet av den siste måneden har kontaktet deg angående en pasients alkoholforbruk? |  |

| **Besvar spørsmålene under i sammenheng med at du i en pasientkonsultasjon enten:**  **Tar initiativ til** en samtale om alkoholforbruk **som del av vanlig rutine**  eller  du spør om alkoholvaner fordi pasienten kan ha **mulig alkoholrelaterte helseproblemer.** |
| --- |

|  | Helt uenig | Uenig | Hverken enig/uenig | Enig | Helt enig |
| --- | --- | --- | --- | --- | --- |
| C1. Jeg har fått **tilstrekkelig trening** i å ta opp alkohol som tema og tilby relevante tiltak | 1 | 2 | 3 | 4 | 5 |
| C2. Jeg har de **nødvendige ferdigheter** for å ta opp alkohol som tema og tilby relevante tiltak | 1 | 2 | 3 | 4 | 5 |
| C3. Jeg er **faglig trygg** når jeg tar opp alkohol som tema og tilbyr relevante tiltak | 1 | 2 | 3 | 4 | 5 |
| D1. Å ta opp alkohol som tema og tilby relevante tiltak er **en del av hverdagen min** som fastlege/allmennlege | 1 | 2 | 3 | 4 | 5 |
| D2. Det er mitt **ansvar** som fastlege/allmennlege å ta opp alkohol som tema og tilby relevante tiltak | 1 | 2 | 3 | 4 | 5 |
| D3. Å ta opp alkohol som tema og tilby relevante tiltak **passer godt med** allmennlegerollen | 1 | 2 | 3 | 4 | 5 |

| **Besvar spørsmålene under i sammenheng med at du i en pasientkonsultasjon enten:**  **Tar initiativ til** en samtale om alkoholforbruk **som del av vanlig rutine**  eller  du spør om alkoholvaner fordi pasienten kan ha **mulig alkoholrelaterte helseproblemer.** |
| --- |

| Jeg føler meg trygg på at jeg kan ta opp alkohol som tema og tilby relevante tiltak, selv om… | Helt uenig | Uenig | Hverken enig/uenig | Enig | Helt enig |
| --- | --- | --- | --- | --- | --- |
| E1 ... pasienten **ikke er motivert** | 1 | 2 | 3 | 4 | 5 |
| E2... pasienten kan oppleve det som **stigmatiserende** | 1 | 2 | 3 | 4 | 5 |
| E3 … det kan være **ubehagelig** for meg | 1 | 2 | 3 | 4 | 5 |
| E4 … det vil føre til **mer arbeid** for meg | 1 | 2 | 3 | 4 | 5 |
| E5 … det kan være **flaut for pasienten eller virke fornærmende** | 1 | 2 | 3 | 4 | 5 |
| E6 … **jeg er bekymret for** at pasienten kanskje ikke tilfredsstiller helsekravene for førerkort | 1 | 2 | 3 | 4 | 5 |

| Hvis jeg tar opp alkohol som tema og tilbyr relevante tiltak vil det … | Helt uenig | Uenig | Hverken enig/uenig | Enig | Helt enig |
| --- | --- | --- | --- | --- | --- |
| F1 … **være bra** for min pasients helse | 1 | 2 | 3 | 4 | 5 |
| F2 … **virke negativt** inn på mitt **forhold** til pasienten | 1 | 2 | 3 | 4 | 5 |
| F3 … ta fokus bort fra **det pasienten egentlig ønsker** å ta opp | 1 | 2 | 3 | 4 | 5 |
| F4 … være spesielt nyttig for mine **eldre pasienter** | 1 | 2 | 3 | 4 | 5 |
| F 5 … være spesielt nyttig for mine pasienter med **psykiske lidelser** | 1 | 2 | 3 | 4 | 5 |
|  |  |  |  |  |  |

| **Besvar spørsmålene under i sammenheng med at du i en pasientkonsultasjon enten:**  **Tar initiativ til** en samtale om alkoholforbruk **som del av vanlig rutine**  eller  du spør om alkoholvaner fordi pasienten kan ha **mulig alkoholrelaterte helseproblemer.** |
| --- |

|  | Helt uenig | Uenig | Hverken enig/uenig | Enig | Helt enig |
| --- | --- | --- | --- | --- | --- |
| G1. **Jeg har et sterkt ønske om** å ta opp alkohol som tema og tilby relevante tiltak når det er aktuelt | 1 | 2 | 3 | 4 | 5 |
| G2. **Jeg planlegger å ta** opp alkohol som tema og tilby relevante tiltak når det er aktuelt | 1 | 2 | 3 | 4 | 5 |
| G3. **Jeg vil**  ta opp alkohol som tema og tilby relevante tiltak til alle pasienter **med psykiske lidelser eller andre kroniske tilstander** | 1 | 2 | 3 | 4 | 5 |
| H1. Jeg har en klar plan for **hvordan** jeg skal ta opp alkohol som tema og tilby relevante tiltak | 1 | 2 | 3 | 4 | 5 |
| H2. Jeg har en klar plan for **i hvilke situasjoner** jeg skal ta opp alkohol som tema og tilby relevante tiltak | 1 | 2 | 3 | 4 | 5 |
| H3. Jeg har en klar plan for **hvor ofte** jeg skal ta opp alkohol som tema og tilby relevante tiltak | 1 | 2 | 3 | 4 | 5 |
| I1. I løpet av den siste måneden **har jeg ofte glemt** å ta opp alkohol som tema og tilby relevante tiltak hos pasienter hvor dette burde ha vært gjort | 1 | 2 | 3 | 4 | 5 |
| I2. Når jeg **fokuserer** på det å ta opp alkohol som tema og tilby relevante tiltak, blir jeg **mindre oppmerksom** på det pasienten **egentlig ønsker å ta opp** | 1 | 2 | 3 | 4 | 5 |
| J1. Jeg **har** **nok** **tid og ressurser** til å ta opp alkohol som tema og tilby relevante tiltak | 1 | 2 | 3 | 4 | 5 |
| J2. Jeg **kan skaffe relevant hjelp** for pasientene mine **i kommunen** om de trenger det | 1 | 2 | 3 | 4 | 5 |
| J3. Jeg **kan skaffe relevant hjelp** for pasientene mine **i spesialisthelsetjenesten** om de trenger det | 1 | 2 | 3 | 4 | 5 |

| **Besvar spørsmålene under i sammenheng med at du i en pasientkonsultasjon enten:**  **Tar initiativ til** en samtale om alkoholforbruk **som del av vanlig rutine**  eller  du spør om alkoholvaner fordi pasienten kan ha **mulig alkoholrelaterte helseproblemer.** |
| --- |

|  | Svært uenig | Uenig | Hverken enig/uenig | Enig | Svært enig |
| --- | --- | --- | --- | --- | --- |
| K1. **Medarbeiderne på kontoret mitt støtter** arbeidet med å ta opp alkohol som tema og tilby relevante tiltak | 1 | 2 | 3 | 4 | 5 |
| K2. De **fleste i mitt profesjonelle nettverk** (erfarne kolleger, veiledere, medlemmer i kollegagruppen/veiledningsgruppen) **støtter** det å ta opp alkohol som tema og tilby relevante tiltak | 1 | 2 | 3 | 4 | 5 |
| K3. **Positive holdninger blant mine praksiskolleger stimulerer meg** til å ta opp alkohol som tema og tilby relevante tiltak | 1 | 2 | 3 | 4 | 5 |

| Å ta opp alkohol som tema og tilby relevante tiltak er noe jeg … | Svært uenig | Uenig | Hverken enig/uenig | Enig | Svært enig |
| --- | --- | --- | --- | --- | --- |
| L1 … gjør **automatisk** | 1 | 2 | 3 | 4 | 5 |
| L2 … gjør **uten at jeg må minne meg selv på det** | 1 | 2 | 3 | 4 | 5 |
| L3 … gjør **uten at jeg tenker over** det | 1 | 2 | 3 | 4 | 5 |
| L4 … begynner å gjøre **før jeg er bevisst på** at jeg gjør det | 1 | 2 | 3 | 4 | 5 |

A = Knowledge
B = Medical Practice
C = Skills
D = Social/professional role and identify (identity?)
E = Beliefs about capabilities
F = Beliefs about consequences
G = Intentions
H = Goals
I = Memory, attention and decision processes
J = Enviromental context and resources
K = Social influences
L = Behavioural regulation

Tusen takk, du er nå ferdig med skjemaet!

kommentarer til skjemaet:

Hvis du har spørsmål om studien, kontakt:

Researcher details

**Supplementary file 7**. Themes and illustrative quotes relating to the acceptability and feasibility of the educational outreach visits

| **Question 1: How acceptable and feasible are educational outreach visits for embedding pragmatic case finding in practice?** | **Key findings** | **Illustrative quotes** |
| --- | --- | --- |
| *Theme 1: Relevance and learning experience* | - Content was seen as relatable and clinically useful, especially concrete examples and evidence. - Participants wanted more interactive, hands-on practice (e.g., Motivational Interviewing, role-play) and clearer guidance for homework to support skill development. | *“The* ***relatable clinical examples and evidence*** *helped bridge the theory with everyday practice.” [GP 1, Practice 4]*  *“It might’ve been even better if the practical tools like Motivational Interviewing were* ***practically oriented****—exercises or interactive modes in Zoom like breakout rooms and Mentimeter.” [GP 2, Practice 2]*  *“The first time it was quite defined… on other occasions it would have helped to have it* ***written down****.” [GP 2, Practice 3]* |
| *Theme 2: Engagement and delivery format* | - Engagement dipped with long sessions, late-day timing, and long intervals; some valued online convenience, but many found virtual sessions less interactive. COVID-19 delays disrupted continuity. | *“We arrive at* ***half past three after work****, so it’s not exactly the brightest part of the day. I zoned out quite a few times.” [GP 1, Practice 1]*  *“The course was* ***postponed by several months*** *because of Covid.” [GP 2, Practice 3]* |
| *Theme 3: Facilitator rapport and support* | - The lead facilitator’s general practitioner background improved relevance and credibility; additional contributors (e.g., digital expert, lived experience) were appreciated. | *“He was* ***one of us****—he knows our routines and the challenges we face.” [GP 1, Practice 4]*  *“It was just, he was just very* ***good at conveying (his knowledge)****, and he had good knowledge (of the subject). [GP 2, Practice 3]*  *“I thought it was good with the* ***user representative, that was good information from that side****. [GP1, Practice 1]* |

**Supplementary file 8**. Themes and illustrative quotes relating to changes in practitioners’ approach to addressing alcohol consumption

| **Question 2: How does embedding PCF affect general practitioners’ (GPs) approaches to discussing alcohol with patients?** | **Sub-themes and key findings** | **Illustrative quotes** |
| --- | --- | --- |
| *Theme 1: Understanding alcohol’s health impact* | *Broadening awareness*   - Education in pragmatic case findings (PCF) expanded GPs understanding of alcohol’s effects beyond addiction. | “*What I, appreciated the most was that, over time, it has* ***changed my attitude*** *maybe to addressing alcohol problems and not just to think about addiction and damaging abuse in a more usual sense, but to have* ***more focus on alcohol’s connection to health problems****.*” [GP 3, Practice 3] |
|  | *Managing chronic diseases*   - A shift towards a more holistic, health-oriented approach to addressing alcohol in general practice. | “*I think the... the pragmatic case-finding makes it so you can bring it up with patients who you wouldn’t think have a... the use isn’t necessarily risky on its own, but who may have medical conditions****,*** *eh... diabetes, or high blood pressure, are overweight, things like that...* ***where you think that talking about it, and maybe... reducing it a bit, can actually better their medical condition*** *and mean they can use less medicine. Which patients are generally very interested in.*” [GP 2, Practice 4] |
| *Theme 2: Recognising hidden cases* | *Sustaining awareness of hidden cases*   - Need for staying alert to less obvious cases. | “*There you will always identify those extreme cases, but the ones where it* ***looks like a completely normal person*** *then aha, this you hadn’t known about. Those are maybe the ones where you can actually make the biggest change with as well, because they are* ***probably motivated*** *because they are well functioning people with one small problem.*” [GP 1, Practice 2] |
|  | *Using tools and monitoring*   - Objective metrics provided a more preventive, data-driven approach to addressing alcohol, which facilitated sustained attention to alcohol-related health concerns. | “*But I also think that the most important contribution the course made was the awareness that you can actually bring up alcohol related questions without there being any very large alcohol related damage. And also that one can* ***use liver measurements*** *which are high within, within reference points, they can be used as a type of monitoring for how it’s going.*” [GP 1, Practice 3] |
| *Theme 3: Normalising alcohol discussions* | *Reducing stigma and focusing no moderation*   - PCF enabled non-judgmental conversations that normalised alcohol discussions and encouraged realistic change. | “*It’s not necessarily abstinence, and that sort of thing, but that it’s possible to... it’s possible to* ***think that reduction*** *is also a good... a good measure you can take. Because patients get frightened when you talk about smoking because they think that it’s, no, about alcohol, because then it becomes like it was for smoking. That “even one is not ok”. It’s... it’s a lot more... less gradual than quitting smoking*.” [GP 1, Practice 4] |
|  | *Building communication skills*   - Motivational interviewing (MI) allowed GPs to open conversations around alcohol more comfortably. | “I think it was very **inspiring to see those videos** [on MI], I have to say that, even though as D1 said at the beginning, it becomes in a way…maybe it won’t always be like, like it is, because we have people who come in with very many different problems, but anyway it’s ins**piring how elegantly it’s possible to talk about alcohol.** I think so.” [GP 3, Practice 3] |
| *Theme 4: Changing clinical habits* | *Balancing motivation and challenges*   - GPs felt more motivated to address alcohol but found changing entrenched routines slow and difficult. | *We’re in the starting blocks, I thought. I think it’s more like a signal to change habits when you‘ve been working for a long time and in a certain way. So suddenly you have to do things differently and it’s about your attitudes, about practicing bringing something up in a new way, so that* ***takes a very long time to become automatic****. And even though I say that I’m very motivated, that I’m doing something. It’s a long way from automatic. And I would like it to be, and I believe that if it’s going to be, then I think we need someone to remind us about some of it after a while.*” [GP 3, Practice 3] |
|  | *Seizing routine opportunities*   - Bringing up alcohol during routine situations facilitated the formation of a habit to address alcohol. | “*You have a series of questions which, after a while, become natural to ask the patients with chronic illness. And it’s often possible to* ***add alcohol as something you simply talk*** *about. As a part of what’s normal to discuss when you, for example, talk with a diabetic patient about how it’s going, right?”* [GP 1, Practice 4] |
